# Chemical constituents and free radical scavenging activity of corn pollen collected from *Apis mellifera* hives compared to floral corn pollen at Nan, Thailand

**DOI:** 10.1186/1472-6882-12-45

**Published:** 2012-04-18

**Authors:** Atip Chantarudee, Preecha Phuwapraisirisan, Kiyoshi Kimura, Masayuki Okuyama, Haruhide Mori, Atsuo Kimura, Chanpen Chanchao

**Affiliations:** 1 Program of Biotechnology, Faculty of Science, Chulalongkorn University, 254 Phayathai Road, Bangkok, 10330, Thailand; 2 Department of Chemistry, Faculty of Science, Chulalongkorn University, 254 Phayathai Road, Bangkok, 10330, Thailand; 3 Honeybee Research Group, National Institute of Livestock and Grassland Science, Ibaraki, 305-0901, Japan; 4 Division of Applied Bioscience, Graduate School of Agriculture, Hokkaido University, Sapporo, 060-8589, Japan; 5 Department of Biology, Faculty of Science, Chulalongkorn University, 254 Phayathai Road, Bangkok, 10330, Thailand

**Keywords:** *Apis mellifera*, Bee corn pollen, Floral corn pollen, DPPH, Free radical scavenging activity, Nutritional components

## Abstract

**Background:**

Bee pollen is composed of floral pollen mixed with nectar and bee secretion that is collected by foraging honey (*Apis* sp.) and stingless bees. It is rich in nutrients, such as sugars, proteins, lipids, vitamins and flavonoids, and has been ascribed antiproliferative, anti-allergenic, anti-angiogenic and free radical scavenging activities. This research aimed at a preliminary investigation of the chemical constituents and free radical scavenging activity in *A. mellifera* bee pollen.

**Methods:**

Bee pollen was directly collected from *A. mellifera* colonies in Nan province, Thailand, in June, 2010, whilst floral corn (*Zea mays* L.) pollen was collected from the nearby corn fields. The pollen was then sequentially extracted with methanol, dichloromethane (DCM) and hexane, and each crude extract was tested for free radical scavenging activity using the DPPH assay, evaluating the percentage scavenging activity and the effective concentration at 50% (EC_50_). The most active crude fraction from the bee pollen was then further enriched for bioactive components by silica gel 60 quick and adsorption or Sephadex LH-20 size exclusion chromatography. The purity of all fractions in each step was observed by thin layer chromatography and the bioactivity assessed by the DPPH assay. The chemical structures of the most active fractions were analyzed by nuclear magnetic resonance.

**Results:**

The crude DCM extract of both the bee corn pollen and floral corn pollen provided the highest active free radical scavenging activity of the three solvent extracts, but it was significantly (over 28-fold) higher in the bee corn pollen (EC_50_ = 7.42 ± 0.12 μg/ml), than the floral corn pollen (EC_50_ = 212 ± 13.6% μg/ml). After fractionation to homogeneity, the phenolic hydroquinone and the flavone 7-O-*R*-apigenin were found as the minor and major bioactive compounds, respectively. Bee corn pollen contained a reasonably diverse array of nutritional components, including biotin (56.7 μg/100 g), invert sugar (19.9 g/100 g), vitamin A and β carotene (1.53 mg/100 g).

**Conclusions:**

Bee pollen derived from corn (*Z. mays*), a non-toxic or edible plant, provided a better free radical scavenging activity than floral corn pollen.

## Background

Bee pollen is collected by foraging honey bees (*Apis* sp. including *A. mellifera*) and stingless bees [1]. It is a combination of principally floral pollen mixed with some nectar or honey, enzymes, wax and bee secretion. The pollen mixture is transported as a small pellet in the pollen basket of the forager’s hind legs to the bee hive where it is stored and used as a food source for the bee larvae [2]. Bee pollen can be considered nutritional, since it contains essential substances, such as carbohydrates, proteins, amino acids, lipids, vitamins, mineral substances and trace elements [3], but its typically low concentration in individual flowers makes it not worthwhile as a mainstay food supply for mammals and most large animals.

The main bioactive compounds reported from bee pollen are phenolic compounds, and specifically quercetin, kaempferol, caffeic acid [4] and naringenin [5]. In addition, the chemical composition of bee pollen depends mainly on which plants are used as pollen sources by the collecting bees, and so are likely to vary according to pollen availability within the foraging colonies range as well as by pollen (flower) selection by the bee species. Thus, bee pollen would be expected to show regional, seasonal and bee species specific variations; consequentially, the chemical composition and bioactivity are likely to vary accordingly. For example, bee pollen in one study site in Brazil was found to mainly be comprised of the pollen from *Scoparia dulcis* L. and *Senna obtusifolia* L. and had the main bioactive chemical components of *p*-hydroxycinnamic acid, dihydroquercetin, isorhamnetin, luteolin and quercetin [6]. That from another site in Portugal was mainly derived from *Salix atrocinerea, Erica AustraliaRaphanus raphanistrum* and *Eucalyptus globules*, with the different principal bioactive chemical components of kaempferol-3-neohesperidoside, quercetin-3-rhamnoside, myricetin-3-galactoside, kaempferol-3-sophoroside, quercetin-3-sophoroside, tricetin, myricetin and luteolin [7].

Globally bee pollen has been reported to provide a diverse array of bioactivities, such as antiproliferative, anti-allergic, antibiotic, antidiarrheic and antioxidant activities [8-10]. In addition, Eraslan et al. [11] reported that bee pollen, in which the dominant pollen was from *Brassica napus* L., could detoxify propoxur, a broad spectrum carbamate insecticide, in experimental rats. Note, however, that was well as the differences between bee pollen samples, the active compounds reported will also reflect variations in the actual bioactivities screened for and in the methodology utilized for screening for them as well as variations in the extraction and enrichment of the compounds.

Free radicals are compounds or an ion that has an electron donor and a molecule of oxygen, such as O^∙^_2_^-^, HO^∙^, ROO^∙^, H_2_O_2_, in the center of the structure [12]. The most common free radicals in biological systems are reactive oxygen species (ROS), and these serve as a connection among signals inside the cells involved in stress responses, cell proliferation, aging and cancer [13]. An excess amount of free radicals can cause damage or death to cells and can lead to many diseases, such as cancer, cataract formation, age-related and muscular degeneration, atherosclerosis, cardiac ischemia, Parkinson’s disease, gastrointestinal disturbance, aging and rheumatoid arthritis [14-16]. In addition, too high a free radical level inside the body has been shown to affect low density lipoprotein (LDL) and to induce protein and DNA damage [17]. Thus, finding new suitable antioxidant agents is still important.

Antioxidant agents have been successfully isolated directly from plants, such as flavonoids, quercetrin (quercetin-3-O-rhamnoside), rutin (quercetin-3-O-rutinoside) and quercetin from *Solidago microglossa *[18], methyl 3,5-dicaffeoyl quinate and 3-O-feruloylquinic acid from *Kalopanax pictus *[19], and flavanones and hydroxycinnamic acid derivatives (polymethoxyflavones and furocoumarin) from citrus fruits in Cyprus [20].

Besides directly from plants, antioxidant agents can also be obtained or found in bee pollen, a source of mainly plant origin. For example, Silva et al. [6] showed that the chemical constituents in the bee pollen of the stingless bee, *Melipona subnitida*, in Brazil had free radical scavenging activity. The bee pollen was largely collected from the pollen of *Mimosa gemmalata* (a plant in the Mimosaceae family) and a plant in the Fabaceae family. Seven active compounds were found, namely naringenin, isorhamnetin, D-manitol, *β*-sitosterol, tricetin, selagin and 8-methoxiherbacetin. These anti-oxidant chemical constituents have also been found in the bee pollen from *A. mellifera *[4].

In addition, the bioactivities of bee pollen have been reported to depend on the geographic region, harvesting period and seasons, as expected and outlined above. Leja et al. [21] reported that the total antioxidant activity, expressed as the percentage of the inhibition of lipid peroxidation, varied significantly among different pollen types. Bee pollen from *Pyrus communis, Malus domestica, Taraxacum officinale, Aesculus hippocastanum, Robinia pseudoacacia, Phacelia tanacetifolia* and *Sinapis alba* provided a total antioxidant activity of greater than 60%. Moreover, other external factors, such as the solvent used in the extraction, and the extraction and pollen storage methods also play an important role in the bioactivities obtained and reported. For example, Negri et al. [22] reported that the methanol extract of untreated bee pollen, bee pollen frozen at −18 °C and bee pollen frozen and then dried presented a significantly different antioxidant activity, with that prepared from pollen that was frozen and then dried being the most active. However, whether this reflects changes in the relative extraction efficiencies or changes in the actual chemical composition, such as from susceptibility to biotic chemical reactions, like enzymic modification, or abiotic ones like oxidation and photodegradation, is unknown.

That the bioactive chemical constituents in bee pollen could be an alternative source for free radical scavenging activity led to our interest in studying the bee pollen of *A. mellifera* in Nan, Thailand. The sample was collected in Nan province because of the unique or typical geography and botanical diversity of the region and so potential diversity of pollen available for bees. However, the region also has commercial agriculture including nearby monoculture corn (*Zea mays* L.) fields which turned out to be significant. Nevertheless, the bee pollen was collected and sequentially extracted with three solvents of decreasing polarity before using bioactivity guided fractionation to yield pure bioactive components. These pure active compounds were then analyzed for their formula structure by NMR. The origin of the pollen in the bee pollen was evaluated by morphology using light and scanning electron microscopy (SEM). The nutritional components in bee pollen were also assayed in order to promote its consumption. The benefit of this work might be that new active anti-oxidant compounds could be obtained and might be developed to be an anti-oxidant agent useful in the pharmaceutical industry. Finally, this may help promote the bee industry in Thailand and so bring an increased income to bee farmers.

## Methods

### Sample collection

Bee pollen was directly collected from a pollen trap at the entrance of *A. mellifera* hives (about 80–100 hives) in Chedeechai subdistrict, Pua district, Nan province, Thailand, in June, 2010. The freshly harvested bee pollen was dried in an oven at 40 °C overnight and then stored at room temperature (RT) (25 °C) until use. Corn (*Z*. *mays* L.) was the dominant plant species surrounding the sampling site and was found to be the principal pollen component of the bee pollen. Floral corn pollen was collected, dried, and stored at RT until used.

### Pollen morphology analysis by scanning electron microscopy (SEM)

Both the collected bee pollen and corn pollen were sent to the Scientific and Technological Research Equipment Centre (STREC) of Chulalongkorn University for morphological analysis. In addition, light microscopy based examination of the pollen grains was performed in comparison to the standard Palynotheca reference work and a standard set of reference pollen samples on slides.

### Extraction

The pollen extraction procedure followed that reported by Umthong et al. [23] and Najafi et al. [24]. Bee pollen (420 g) was stirred with 80% (v/v) methanol (2,400 ml) at 100 rpm, 15 °C for 18 h and then clarified by centrifugation at 5,500 x g, 4 °C for 15 min. The supernatant (methanolic extract) was harvested and the solvent evaporated under reduced pressure and a maximum temperature of 40–45 °C to leave the crude methanol extract of bee pollen (CMEb). The pellet (methanol extracted bee pollen residue) was then extracted with 2,400 ml of dichloromethane (DCM) under the same conditions as above, and the harvested solvent evaporated to yield the crude DCM extract of bee pollen (CDEb). Finally, the pellet (methanol and DCM extracted pollen residue) was extracted with hexane (2,400 ml) in the same manner to leave the crude hexane extract of bee pollen (CHEb). All three crude extracts were kept in the dark at −20 °C until they were tested for the free radical scavenging activity by the 1,1-diphenyl-2-picrylhydrazyl (DPPH) assay or further enriched (see below).

### Chromatography

#### Quick column chromatography

The crude extracts were first fractionated by vacuum quick silica gel 60 (Merck) column chromatography (VCC, 500 ml in size). Two grams of the crude extract (CMEb, CDEb or CHEb) was dissolved in DCM (3 ml) and combined with 10 g of rough silica gel 60, left to dry and then sprinkled evenly over the surface of the packed VCC. This was then covered with silica gel, a piece of filter paper and cotton, respectively, in order to protect the crude layer. The packed chromatography column was then eluted with a step gradient mobile phase comprised of 750 ml of 3:1 (v/v) DCM: hexane, 500 ml of DCM, 1250 ml of 1:19 (v/v) methanol: DCM, 1250 ml of 1:9 (v/v) methanol: DCM, 4000 ml of 1:4 (v/v) methanol: DCM and 2000 ml of methanol, collecting 250 ml fractions. In order to attain that flow rate an aspirator was used. Each 250 ml fraction was collected in a 500 ml flask and the solvent evaporated under reduced pressure and a maximum temperature of 40–45 °C. The pattern of chemical compounds in each fraction was tested by thin layer chromatography (TLC) (see below) and fractions providing the same profile pattern of chemical compounds across the TLC plates were pooled together. Each resultant (pooled) fraction was then assayed for free radical scavenging activity using the DPPH assay (see below). Active fractions were further purified by Sephadex LH-20 size exclusion and silica gel 60 adsorption chromatography.

#### Sephadex LH-20 size exclusion chromatography

Sephadex LH-20 gel (100 g, GE Healthcare Bio-sciences AB), preswollen in absolute methanol (500 ml) overnight, was used to form the column (150 ml in size with an additional 100 ml head height). Each active fraction was dissolved in absolute methanol until it was not too viscous and then loaded onto the column and eluted with 500 ml of 100% (v/v) methanol (500 ml) collecting 2.5 ml fractions. Fractions were tested for different chemical compositions and bioactivity by TLC, the latter being performed by spraying the developed TLC plate with 0.2% (w/v) DPPH in methanol (see below).

#### Adsorption chromatography

Silica gel 60 (90 g) was mixed with 200 ml of 1:49 (v/v) methanol: DCM and used to pack the 150 ml column. Each active (pooled) fraction obtained from the Sephadex LH-20 chromatography was mixed with silica gel 60 (10 g), allowed to dry, and placed evenly on top of the packed gel in the column. The column was eluted with 500 ml of 1:49 (v/v) methanol: DCM, collecting 2.5 ml fractions. The purity and bioactivity of all fractions was tested by TLC and DPPH spraying.

#### Thin layer chromatography (TLC)

TLC plates (Si_60_/F_254_ as the immobile phase) were prepared as 5 x 5 cm sheets. Each respective sample was applied onto each of eight replicate plates, allowed to dry and then one-dimension resolved, one replicate plate per mobile phase solvent (hexane, 1:1 (v/v) DCM: hexane, 3:1 (v/v) DCM: hexane, DCM, 1:19 (v/v) methanol: DCM, 1:9 (v/v) methanol: DCM, 3:17 (v/v) methanol: DCM and 1:4 (v/v) methanol: DCM). The TLC plate was then allowed to dry and the conjugated compounds on the plate were visualized under short and long wavelength ultraviolet light at 254 and 356 nm.

### Free-radical scavenging activity

#### 1, 1-Diphenyl-2-picrylhydrazyl (DPPH) assay

The assay for free radical scavenging activity using the DPPH assay followed the procedure reported by Chen et al. [25] using five different concentrations of the test fractions/sample (0, 10, 50, 100 and 400 μg/ml) in methanol. For each concentration, 50 μl of the test fraction was mixed with 50 μl of 0.15 mM DPPH in methanol in a 96 well-plate, incubated at RT for 1 h in the dark, and then the absorbance was measured at 517 nm by a microplate reader (Sunrise, Tecan). Ascorbic acid (vitamin C) at 10–1000 μg/ml was used as the standard reference. Experiments were performed in triplicate. The free radical scavenging activity was calculated as follows:

(1) $$\text{Percentage of free radical scavenging activity }=\frac{\left(\text{ABS control }–\text{ ABS sample}\right)\times \;100}{\text{ABS control}}$$

where ABS control and ABS sample are defined as the absorbance at 517 nm of the control and sample, respectively.

#### The effective concentration at 50% (EC_50_)

The effective concentration at 50% (EC_50_) was obtained by graphical extrapolation after plotting the percentage of free radical scavenging activity (Y-axis) against the concentration of each sample (X-axis) and using regression analysis to plot the best fit line.

#### DPPH spray

To evaluate the free radical scavenging activity of each fraction or component on the resolved TLC plate, in qualitative terms only of strong, moderate, weak or no activity, the method reported by Braca et al. [26] was followed. In brief, the TLC plates were sprayed with 0.2% (w/v) DPPH in methanol and left. An active spot would turn from violet to yellow.

### Chemical structure analysis

An evaporated pure fraction was sent for the structure analysis at Department of Chemistry, Faculty of Science, Chulalongkorn University. Briefly, the evaporated sample was dissolved in an appropriate deuterated solvent (methanol-D4, CD_3_OD, Merck) at the ratio of 2–5 mg of compound to 500 μl of the deuterated solvent, mixed well and then the NMR spectra was recorded on a Varian Mercury^+^ 400 operated at 400 MHz for ^1^ H and 2D NMR (COSY, HSQC and HMBC) or at 100 MHz for ^13^ C nuclei. Tetramethylsilane (TMS) was used as an internal standard. The value of the chemical shift in δ (ppm) was assigned with the reference to the signal from the residual protons in the deuterated solvent.

### Free radical scavenging of *Z. mays* pollen

Corn pollen was collected from stamens and sequentially extracted by methanol, DCM and hexane to yield the crude methanolic (CMEp), DCM (CDEp) and hexane (CHEp) extracts, and the evaluation of the free radical scavenging activity in each extract were performed, all as outlined above for bee pollen.

### Nutritional composition analysis

Bee pollen was sent to the Central Laboratory (Thailand) Co., Ltd., Kasetsart University, Thailand, for evaluation of its composition in terms of the ash, biotin, calories, calories from fat, carbohydrate, crude fiber, fat, protein, folic acid, invert sugar, moisture, vitamin complex, fructose, glucose, sucrose, maltose and lactose contents. In addition, a sample was sent to the Institute of Food Research and Product Development, Kasetsart University, Thailand, for co-enzyme Q10 analysis. Later, the obtained data was compared to the reported nutritional composition of bee pollen from other plant origins and countries.

## Results

### Pollen morphology

**Figure 1:**
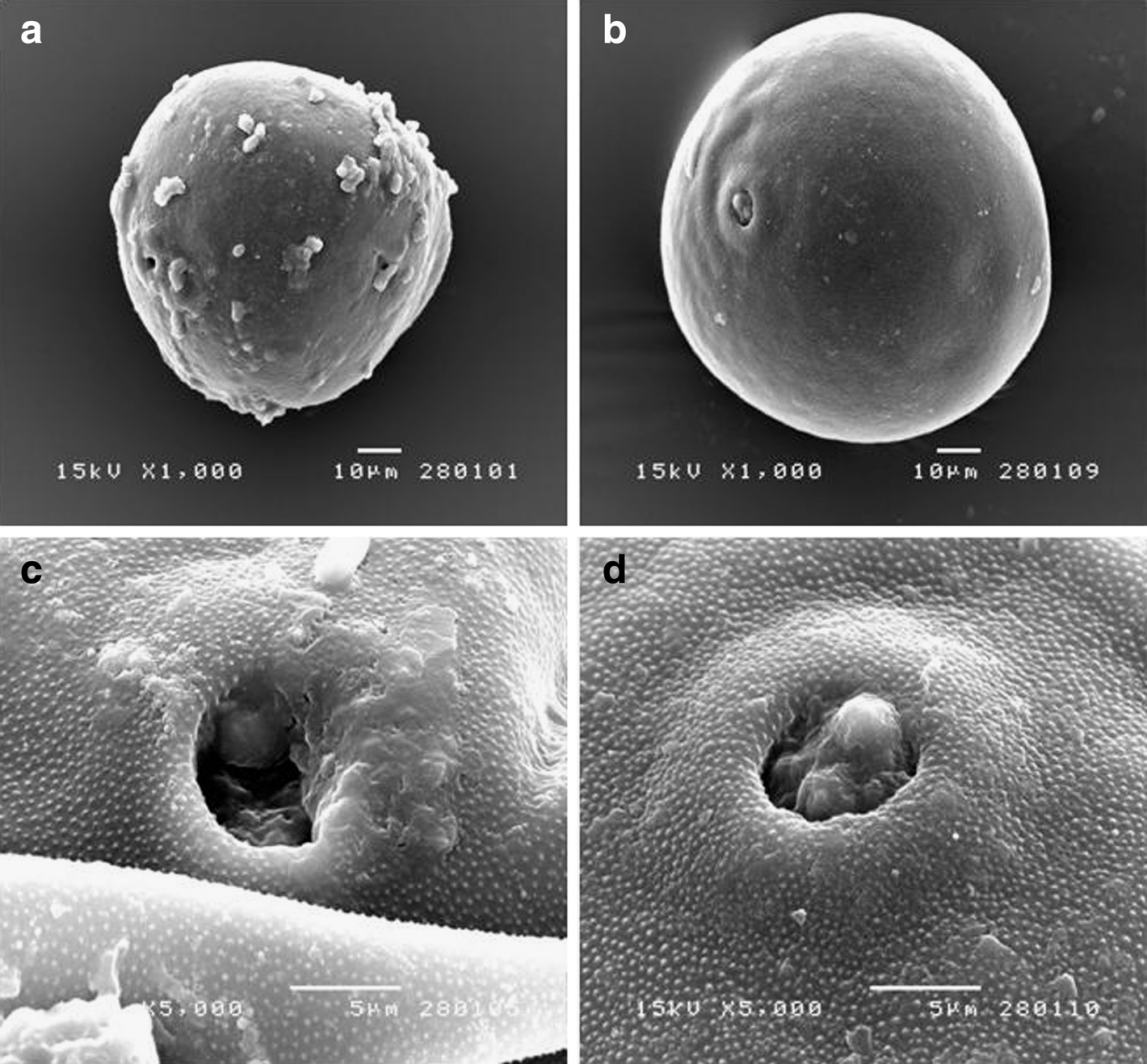
**Comparison of the (A, C) *A. mellifera *harvested bee pollen with that of (B, D) corn pollen by SEM showing (A, B) the external morphology (1000 x magnification) and (C, D) the germination pore and ornamentation of the pollen exine (5000 x magnification). **Micrographs shown are representative of those seen from 10 independent pollen grains.

Considering the bee pollen morphology in terms of its shape and color, as observed by light microscopy, when compared to the morphology of different plant pollens from a set of standard slides and the recorded figures in the Palynotheca book, the bee pollen appeared to be mainly (>95% from randomly examined samples) comprised of corn pollen. In agreement, SEM analysis revealed that the ornamentation of the exine and germination pore of the bee pollen were very similar to that of the corn pollen, as were the germination pore and the surface of outer wall (exine) morphology (Figure 1). Thus, the principal botanical origin of the collected *A. mellifera* bee pollen was determined to be from corn. Although situated in a biodiverse forest region, this is not unrealistic given the hives were situated at the time near agricultural corn plantations which were in flower, and so the bees had likely foraged for pollen in the wealth of corn tassels rather than the more sparse (and diverse) forest flowers.

Consequentially, pollen from the nearby flowering corn plantation was harvested and extracted along with the bee pollen for a direct comparison.

### Crude extracts of bee pollen and corn pollen

The sequential extraction of the bee pollen from Nan with methanol, DCM and hexane gave extracts of different yields and appearance (Table 1). Since the yields match the extraction order and extractions were not performed in the reverse sequential order, or with each solvent only, it is not possible to reliably ascribe each yield as representing the proportion of polar or non-polar components in the pollen, as compounds may be differentially soluble in more than one solvent. However, by far the highest (14.7-fold) yield was obtained in the CMEb, which tends to support the existence of a wealth of moderately polar compounds in the bee pollen. Note, however, that we did not extract the pollen with water for strongly polar compounds. The CMEb extract was also sticky, perhaps representative of the fatty acids, glycoproteins and mucopolysaccharides in the bee pollen.

**Table 1:** The weight and the character of the three crude solvent extracts from bee pollen and corn pollen

**Crude extract**^**a**^	**Yield**^**b**^ **(g)**	**Yield**^**c**^ **(% of pollen sample)**	**Appearance**
*Corn bee pollen extracts*			
CMEb	35.29	8.40	Dark brown sticky solid
CDEb	2.40	0.57	Dark brown solid
CHEb	18.13	4.32	Yellow solid
*Floral corn pollen extracts*			
CMEp	9.02	2.15	Dark brown solid
CDEp	4.82	1.15	Brown solid
CHEp	7.87	1.87	Dark green oil/wax

^a^CMEb, CDEb and CHEb stand for the crude methanol, DCM and hexane extracts of the bee pollen, while CMEp, CDEp and CHEp stand for the crude methanol, DCM and hexane extracts of the floral corn pollen.

^b,c^The yield of each crude extract as ^b^the net weight (g) and ^c^the proportion (% (w/w)) of the total starting pollen mass.

The crude methanol, DCM and hexane extracts of the corn pollen (CMEp, CDEp and CHEp, respectively) were prepared as per the bee pollen. The yields obtained and appearance of each crude extract varied (Table 1), but showed clear differences in both the yields obtained within and between solvents and their appearances compared to that seen in the extracts from bee pollen (Table 1), despite being principally from the same plant source (*Z. mays*).

### Free radical scavenging activity of crude extracts

**Figure 2:**
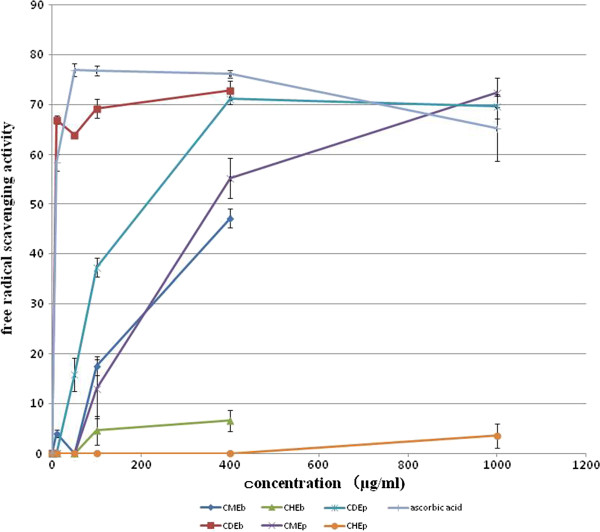
**Free radical scavenging activity of the crude solvent extracts of bee pollen and corn pollen. ***A. mellifera *bee pollen and corn pollen were extracted sequentially with methanol (CMEb, CMEp), DCM (CDEb, CDEp) and hexane (CHEb, CHEp) prior to assaying for free radical scavenging activity using the DPPH assay in comparison to the reference standard of ascorbic acid. Data are shown as the mean ± 1 SEM, derived from three independent repeats. Means with a different letter are significantly different (*p *< 0.05).

Crude bee pollen extracts (CMEb, CDEb and CHEb) and floral corn pollen extracts (CMEp, CDEp and CHEp) were tested for their free radical scavenging activity using the DPPH assay, with the obtained absorbance being converted to the mean (± S.E.M.) percentage scavenging activity (Figure 2).

The highest free radical scavenging activity at all four concentrations was found with the CDEb (≥ 63%), and this was close to that of the ascorbic acid reference standard. In contrast, CMEb showed essentially no activity at 10 or 50 μg/ml, requiring 100 or 400 μg/ml for a weak or moderate activity, respectively. CHEb, on the other hand, showed essentially no activity at all. As a reference, the highest free radical scavenging activity of ascorbic acid (50 μg/ml) was 76.9 ± 1.27%. In terms of the estimated EC_50_ values, the CDEb provided the lowest EC_50_ value, even slightly lower than that of ascorbic acid (Table 2).

**Table 2:** **The EC**_**50**_**values for the free radical scavenging activity of three different crude extracts from bee pollen and corn pollen, plus the reference standard (ascorbic acid) for comparison**

**Sample**	**EC**_**50**_**(μg/ml)**^**ƒ**^	***r***^***2*** **ƒ**^
*Bee corn pollen extracts*		
CMEb	428.6 ± 29.0^b^	ND
CDEb	7.47 ± 0.12^a^*	1
CHEb	>1,000^c^	ND
*Floral corn pollen extracts*		
CMEp	365.2 ± 38.8^bb^	1
CDEp	212.3 ± 13.6^aa^**	1
CHEp	>1000^cc^	ND
Ascorbic acid	8.57 ± 0.24*	1

^**ƒ**^ EC_50_ values were calculated by linear regression (*r*^*2*^ = the correlation coefficient). ND represents no available data. Means within a group of three crude extracts of each sample with a different superscript letter (bee pollen extracts) or pair of letters (floral pollen extracts) are significantly different (p ≤ 0.05; One way ANOVA), whilst a significant difference between groups is indicated by * or **.

As with the bee pollen, the hexane extract of the corn pollen (CHEp) was essentially free of any free radical scavenging activity, whilst the highest activity was seen in the CDEp followed by the CMEp (Figure 2). However, the activity level seen in the CDEp was statistically significant lower than that in the corresponding CDEb from bee pollen at lower, but not at higher, concentrations, with the derived EC_50_ values then being much higher in the corn pollen extracts than in the corresponding bee pollen extracts (Table 2).

### Compound purification

#### Active fractions from quick column chromatography

Since the CDEb provided the best free radical scavenging activity, as indicated by the lowest EC_50_ value, it was selected for further purification using quick column chromatography. A total of 39 fractions were collected but, after pooling fractions with a similar TLC profile across the eight plates (data not shown), seven different fractions (CDEb1–CDEb7) were finally obtained (Table 3) that varied in amount from 5 to 776 mg, which represents a yield of 0.25–38.99% of the CDEb fraction and of 0.001–0.18% of the bee pollen. In general, a higher yield of samples and of darker pigmented appearance was obtained in the latter fractions, eluted with the more polar solvents. Along with the free radical scavenging results, it is then highly likely that the pigments and target compounds should contain some degree of polarity in their structure.

**Table 3:** The weight and character of the seven different active fractions following silica gel quick column chromatography of the DCM extract of bee corn pollen (CDEb)

**Fraction**	**Weight (mg)**	**Yield**^**a**^ **(CDEb)**	**Yield**^**b**^ **(Bee pollen)**	**Character**	**Activity**^**c**^
CDEb1	62	2.58	0.01	Clear white solid	--
CDEb2	42	1.75	0.01	Pale yellow solid	--
CDEb3	6	0.25	0.00	Pale brown solid	--
CDEb4	5	0.21	0.00	Pale brown solid	+
CDEb5	776	32.3	0.18	Dark brown solid	+++
CDEb6	430	17.9	0.10	Sticky, dark brown solid	+++
CDEb7	760	31.7	0.18	Dark brown solid	++

^a,b^Yield as a % of the ^a^CDEb fraction and ^b^of the initial bee pollen mass

^c^Qualitative free radical scavenging activity, as determined by the DPPH assay sprayed onto the TLC plates, where -, +, ++ and +++ represent no, weak, moderate and strong color change (activity).

The free radical scavenging activity, determined by the DPPH assay, of fractions CDEb1-CDEb7 was evaluated and the results are summarized as the percentage of scavenging activity in Table 4. The highest free radical scavenging activity was found in fraction CDEb6, which was higher than the reference standard (ascorbic acid), although reasonable activity was also found in fractions CDEb5 and CDEb7. Essentially no activity was present in fractions CDEb1–3, and only weak activity in CDEb4 at high doses (400 and 1000 μg/ml). The activity of the unfractionated CDEb is also shown for comparison (Table 4), where it was noted to be superior to all of the fractions (CDEb1–7), especially at lower concentrations, suggesting perhaps the possibility of synergism between components that is lost upon their separation, the preferential enrichment of inhibitors in the CDEb1–7 fractions, loss of the more active component(s) during the fractionation or the degradation of the bioactive components during the enrichment procedure. For the estimated EC_50_ values (Table 4), the same trends are noted, except that the values for CDEb5-7 are much higher than that for the ascorbic acid reference standard or the CDEb starting material.

**Table 4:** The percentage scavenging activity of the crude DCM extract of bee pollen (CDEb) and its subfractions (CDEb1 - CDEb7) following silica gel 60 quick column chromatography

**Fraction**	**Percentage scavenging activity**					**EC**_**50**_	***r***^**2**^
	**10 μg/ml**	**50 μg/ml**	**100 μg/ml**	**400 μg/ml**	**1000 μg/ml**	**(μg/ml)**	
CDEb1	3.76 ± 4.27	0.04 ± 2.05	0.31 ± 2.54	0.00	8.29 ± 2.41	>1000	
CDEb2	3.16 ± 1.47	0.00	0.00	0.00	5.92 ± 1.85	>1000	
CDEb3	1.43 ± 2.07	0.1 ± 1.58	0.00	0.00	9.05 ± 0.54	>1000	
CDEb4	0.00	0.00	0.00	14.35 ± 5.91	12.0 ± 0.54	>1000	
CDEb5	5.15 ± 0.7	14.0 ± 3.04	22.5 ± 1.86	59.8 ± 3.98	65.3 ± 0.8	322 ± 27	1
CDEb6	3.69 ± 3.7	19.4 ± 3.80	37.6 ± 1.33	76.7 ± 1.2	78.4 ± 1.44	196 ± 9.9	1
CDEb7	3.34 ± 1.33	6.19 ± 2.13	9.69 ± 0.8	38.3 ± 4.8	58.5 ± 1.71	762 ± 67	1
CDEb	66.9 ± 0.88	68.8 ± 0.47	70.8 ± 0.73	70.1 ± 0.47	61.8 ± 0.7	7.47 ± 0.12	1
Ascorbic acid	58.4 ± 1.7	76.9 ± 1.3	76.8 ± 1.05	76.1 ± 0.72	65.2 ± 6.5	8.57 ± 0.24	1

The reference standard, ascorbic acid, is shown for comparison.

EC_50_ values were calculated by linear regression (*r*^*2*^ represents the correlation coefficient). ND represents no available data. Data are shown as the means ± 1 SD and are derived from 3 independent repeats.

#### Sephadex LH-20 size exclusion chromatography

From the pattern of compounds revealed on the TLC plates (data not shown), the CDEb5 fraction (two distinct compounds) was purer than the others (not separated compounds for CDEb6 and CDEb7) and so fraction CDE5 was selected for further enrichment by Sephadex LH-20 chromatography.

A total of 58 fractions were obtained from CDEb5 after Sephadex LH-20 chromatography, but after pooling of fractions that presented similar compound profiles across the TLC plates (data not shown), six different fractions were obtained (CDEb5-1 to CDEb5-6), ranging from 2.8 to 188.4 mg in yield, or 0.36–24.28% of the CDEb5 fraction and 0–0.04% of the initial bee pollen (Table 5).

**Table 5:** The weight and character of the pooled active fractions derived from the Sephadex LH-20 size exclusion chromatography of the bee pollen derived CDEb-5 fraction

**Fraction**	**Weight (mg)**	**Yield**^**a**^ **(CDEb-5)**	**Yield**^**b**^ **(Bee pollen)**	**Character**	**Activity**^**c**^
CDEb5-1	28.1	3.62	0.01	Brown solid	
CDEb5-2	188.4	24.3	0.04	Dark brown solid	
CDEb5-3	7.8	1.01	0.00	Dark brown solid	
CDEb5-4	3.9	0.50	0.00	Brown solid	
CDEb5-5	2.8	0.36	0.00	Brown solid	++
CDEb5-6	9.9	1.28	0.00	Yellow-green solid	++

^a,b^Yield as a % of the ^a^CDEb5 fraction and ^b^of the bee pollen

^**c**^Qualitative free radical scavenging activity, as determined by the DPPH assay sprayed onto the TLC plates, where -, +, ++ and +++ represent no, weak, moderate and strong color change (activity).

**Figure 3:**
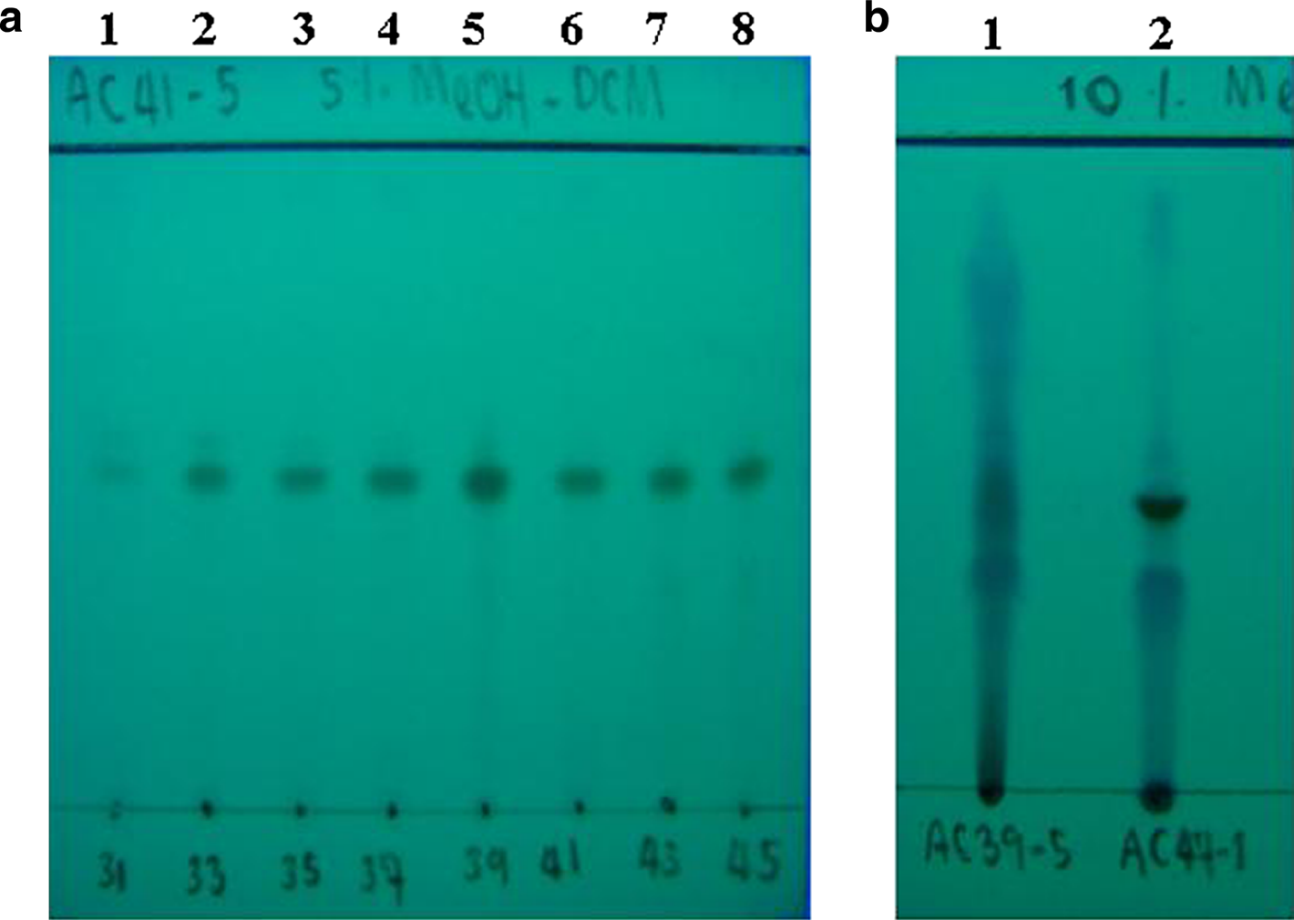
**Representative TLC plates showing the compound profile. **(**A**), Lanes 1–8 contain fractions 31, 33, 35, 37, 39, 41, 43 and 45, respectively, from the Sephadex LH-20 chromatography of CDEb5-5. The potentially pure band of fractions# 33–45 (pooled as compound I) is indicated by an arrow. (**B**), Lane 1 contains CDEb5 while lane 2 contains pooled fractions# 70–109 from CDEb5–6 (compound II, indicated by an arrow). The solvent system used in the shown TLC plates were (**A**) 1:19 (v/v) methanol: DCM and (**B**) 1:9 (v/v) methanol: DCM. TLC plates shown are representative of three replicates.

However, since only a small amount of each fraction (CDEb5-1 to CDEb5-6) was obtained, the free radical scavenging activity was qualitatively assayed by the DPPH spray method on the samples resolved on the TLC plates instead. Fractions CDEb5-5 and CDEb5-6 were the most active (Plates not shown; data summarized in Table 5). The separating pattern of the different compounds on the TLC plates was then considered. In CDEb5-5 they were smears or connected to each other, and so this fraction was further separated by Sephadex LH-20 size exclusion chromatography. For CDEb5–6 each compound was well separated and so silica gel adsorption chromatography was performed. Following the respective fractionation, TLC resolution of the fractions and DPPH spraying of the plates, the same apparent pattern of active compound was obtained from fractions CDEb5–5# 33–45, which were then pooled as compound I (2.2 mg; 78.6% yield) (Figure 3A), whilst fractions #70–109 from CDEb5–6 were pooled to yield compound II (6 mg; 60.6% yield) (Figure 3B).

### Chemical structure analysis

Although Fathiazad et al. [27] used DMSO-d_6_ as the deuterated solvent for running ^1^ H-NMR, it has been suggested that methanol-d4 (CD_3_OD) is more suitable for use with high polarity target samples in small amounts, since the sample could be re-evaporated and re-used. In contrast, DMSO-d_6_ is suitable for very high polarity target samples present in large amounts since the dissolved compound can not be re-used [28,29]. In this research, since only a small amount of compound I was isolated from the bee pollen, CD_3_OD was used.

**Figure 4:**
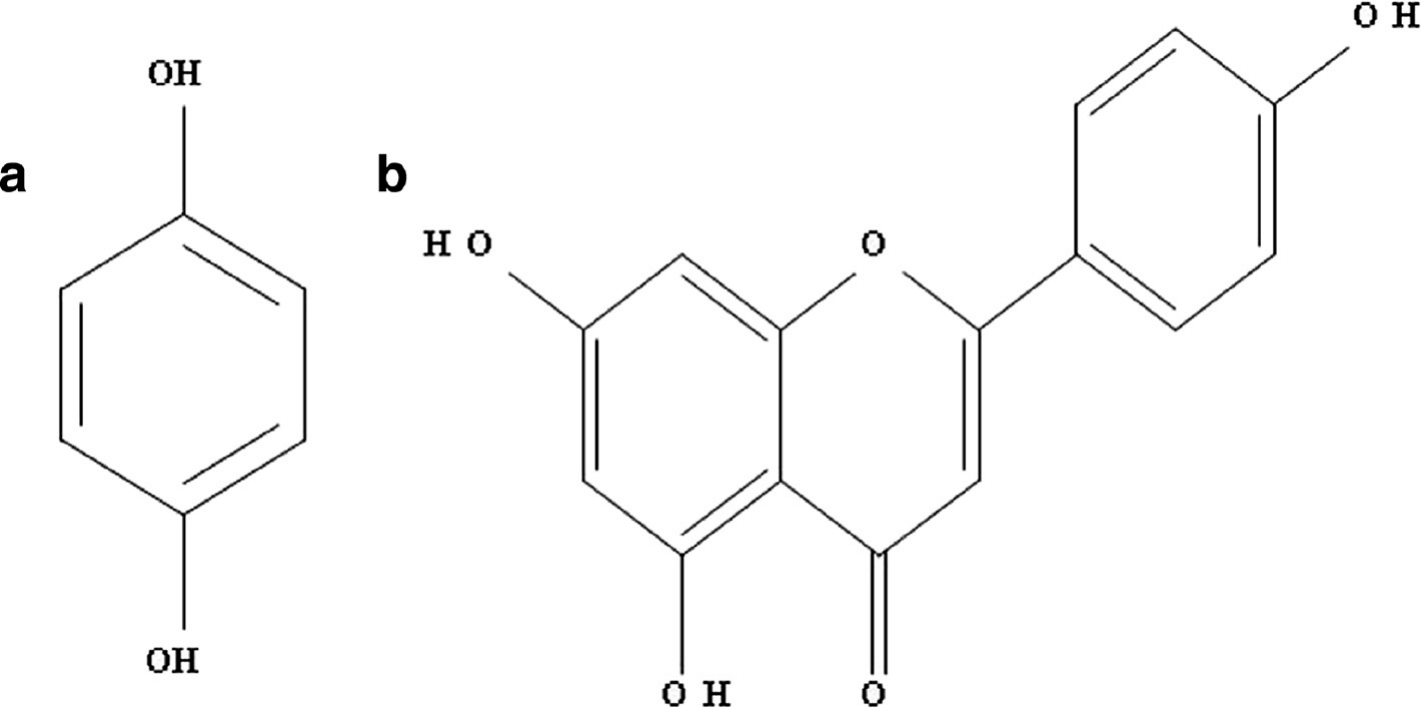
**The formula structure of the two free radical scavenging active compounds in bee pollen: (A) hydroquinone (compound I) and (B) 7-*O*-R-apigenin (compound II).**

Compound I, which was a clear yellow solid, revealed NMR peaks with a chemical shift pattern of ^1^ H-NMR (CD_3_OD, 400 MHz): *δ*7.22 (2 H, d, *J* = 8.8 Hz, H-2 and H-6) and 6.72 (2 H, d, *J* = 8.4 Hz, H-3 and H-5); and ^13^ C-NMR (CD_3_OD, 100 MHz). Thus, compound I was determined to be hydroquinone (Figure 4A).

Compound II, a green-yellow solid, yielded NMR peaks with a chemical shift pattern of ^1^ H-NMR (CD_3_OD, 400 MHz): *δ*_H_ 7.77 (1 H, s, H-3), 7.63 (2 H, d, *J* = 8.8 Hz, H-2′ and H-6′), 6.83 (2 H, d, *J* = 8.4 Hz, H-3′ and H-5′), 6.32 (1 H, s, H-8) and 6.09 (1 H, s, H-6); and ^13^ C-NMR (CD_3_OD, 100 MHz). Thus, it indicated that compound II was 7-0-*R*-apigenin (Figure 4B).

### Nutritional composition

The results are summarized in Table 6. The bee pollen, comprised mainly of corn pollen, could be considered nutritional since it contained a high level of calories, sugar, vitamins, protein, co-enzyme and moderate fat levels. However, its potential allergenic effect and toxicity have yet to be determined.

**Table 6:** **Nutritional composition of bee corn pollen of A.** ***mellifera*** **from Nan province, Thailand**

**Analyzed item**	**Quantity**	**Unit**
**Ash**	2.43	g/100 g
**Biotin**	56.69	μg/100 g
**Calories**	397.16	Kcal/100 g
**Calories from fat**	63.00	Kcal/100 g
**Carbohydrate**	64.42	g/100 g
**Crude fiber**	0.86	g/100 g
**Fat**	7.00	g/100 g
**Folic acid**	54.70	μg/100 g
**Invert sugar**	19.85	g/100 g
**Moisture**	7.03	g/100 g
**Protein**	19.12	g/100 g
**Reducing sugar**	14.11	g/100 g
**Total sugar**	14.71	g/100 g
**Fructose**	7.16	g/100 g
**Glucose**	6.42	g/100 g
**Sucrose**	0.60	g/100 g
**Maltose**	0.53	g/100 g
**Lactose**	< 0.1	g/100 g
**Vitamin A - β carotene**	1,530.4	μg/100 g
**Vitamin B**_**1**_	0.20	mg/100 g
**Vitamin B**_**2**_	0.50	mg/100 g
**Vitamin B**_**3**_**(Niacin)**	7.03	mg/100 g
**Vitamin B**_**5**_	0.39	mg/100 g
**Vitamin B**_**6**_	Not detected	mg/100 g
**Vitamin B**_**12**_	1.87	μg/100 g
**Vitamin E (α-Tocopherol)**	6.21	mg/100 g

## Discussion

Bee pollen is an apicultural product and is usually composed of pollen species from various plants. It is widely consumed for food and nutritional supplements or diets. Thus, it is important to be certain that bees collected the pollen from non-toxic or hyper allergenic plants. Since the pollen morphology of each plant is unique and typical, it can be used to classify the plant source using light and SEM analysis. The morphology of the bee pollen of this study, when examined by light and SEM, was categorized as being mainly (>95%) composed of that from corn. Considering the bee hives (bee pollen sampling site) at the time of collection were located near commercial corn fields in flower this is not unreasonable since the corn would be within the foraging distance of the bees. Thus, it was highly possible that in this collecting period (June) the *A. mellifera* foragers had collected pollen from corn rather than the nearby forest flora. It remains unknown then what the bee pollen would be comprised of, and so its free radical scavenging activity, in other parts of the year when corn pollen was not available for gathering.

Bee pollen could be a preferable source for free radical scavenging activity over that of free corn plant pollen. This is in agreement with the report of Kroyer and Hegedus [30], who found that the bee pollen extract showed a much better antioxidant and free radical scavenging activity than the floral pollen. Among the three different crude extracts, the DCM extract of bee pollen (CDEb) was the best source for the free radical scavenging activity with an EC_50_ value (7.47 ± 0.12 μg/ml), which is close to that for the ascorbic acid standard (8.57 ± 0.24 μg/ml). Considering the medium polarity level of the DCM solvent, then the active compounds in the CDEb would be expected to contain a medium level of polarity in their structure, which was found to be the case in the two isolated compounds in this study (hydroquinone and 7-0-*R*-apigenin). Silva et al. [6] reported that bee pollen extracted by ethyl acetate, which is also a medium polar solvent, revealed a lower EC_50_ value (15.3 ± 0.4 μg/ml) compared to that by hexane and ethanol.

When the CDEb fraction was further fractionated by quick column silica gel 60 chromatography, the seven obtained fractions (CDEb1-CDEb7) all provided a lower free radical scavenging activity than the starting CDEb material at each concentration, with the EC_50_ values of CDEb1-CDEb7 being much higher (> 100 μg/ml) than that for CDEb (7.47 ± 0.12 μg/ml). This could be explained as being due to the presence of strong synergy between components, the loss of the more active components in the fractionation or the preferential enrichment of inhibitors, or the chemical degradation (including by oxidation or photodegradation) of the active components during the enrichment process. Certainly, Kroyer and Hegedus [30] suggested that pollen extracts represent a concentrated mixture of different interacting active compounds.

Nevertheless, after the bee pollen was extracted and purified, two active compounds were still obtained. 7-*O*-R-Apigenin, a flavanoid in the flavones group, was the main active compound, while the phenol hydroquinone was the minor active compound. Accordingly, Ceska and Styles [31] reported the chemical constituents of corn pollen were varieties of flavanoids, such as quercetin, isorhamnetin, kaemferol and flavanol glycoside, although they did not report apigenin.

Apigenin glycosides have been previously reported in *Prosopis juliflora* bee pollen from Mexico [32]. However, in this Thai sample of bee pollen, we found a different derivative (7-*O*-R-apigenin), which is reported for the first time in bee pollen.

The minor active compound found was the phenolic compound, hydroquinone. This has previously been reported to have an antioxidant activity and is added into cosmetics like skin care products [33]. In agreement, Morais et al. [34] reported that the phenolic compounds in the bee pollen from a site in Portugal were the main antioxidant and antimicrobial active compounds.

Although many research papers have recently reported on the chemical composition of bee pollen, none have reported 7-*O*-R-apigenin and hydroquinone yet [4,5,35].

Since this bee pollen likely originally came from corn, the free radical scavenging activity of the crude extracts from both sources were compared. Although the crude DCM extracts of bee pollen (CDEb) and corn pollen (CDEp) showed the highest free radical scavenging activity of the three extracts, CDEb presented a better activity than CDEp with EC_50_ values of 7.41 ± 0.12 and 212.3 ± 13.6 μg/ml, respectively. Thus, bee pollen, which consisted of packed or modified corn pollen, had a better free radical scavenging activity than the natural form of corn pollen. It is not clear how honey bees modify the physical or chemical structure of the pollen by the functional combination of nectar, enzymes and bee secretions when they pack the corn pollen. The derivatized or modified pollen form or active compounds may have a different extraction efficiency, stability to degradation or bioactivity. Regardless, bee pollen collected by *A. mellifera* from (principally) corn plants in Nan province, Thailand, appeared to be an alternative source for free radical scavenging activity, and in the future it may be possible to develop bioactive compounds in bee pollen as antioxidant agents.

Other than the free radical scavenging activity, the nutritional composition of the bee pollen was measured. The quantity of the nutritional compounds, in terms of biotin, crude fiber, folic acid, vitamin B_3_, vitamin B_5_, vitamin B_12_ and co-enzyme Q10, are the first reports of such in bee pollen from Thailand. However, compared to other reports from elsewhere, among the vitamins, this Thai bee pollen sample contained a lower level of α-carotene, β-carotene, vitamin B1 and vitamin E, but higher levels of polysaccharides and sugar than in *Cystus incanus* L. bee pollen from Croatia [4,36]. Moreover, the fat, protein and ash levels were similar to bee pollen from the South of Brazil, where its botanical origin was from the common plant species within the Arecaceae, Asteraceae and Myrtaceae families [4,37].

Overall, the data showed that bee pollen from Nan province, Thailand, during the corn flowering season not only gave a good free radical scavenging activity but it also contained a variety of nutritional supplements. Corn cultivation together with *A. mellifera* cultivation could potentially be transiently (within the time window of the flowering corn crop seasons) managed within the same area as long as other suitable nectar sources were available. Furthermore, it should be possible to apply bee pollen and its extract to food and cosmetic products with the free radical scavenging activity, at least on a limited (so high add on value) scale.

## Conclusions

*Apis mellifera* derived bee pollen, principally collected from corn, in Nan, Thailand, showed a significantly greater free radical scavenging activity than that of the local corn pollen in the same area. The crude DCM extract of the bee pollen (CDEb) revealed a slightly greater free radical scavenging activity than the reference standard (ascorbic acid) and the active fractions of CDEb obtained after purification (CDEb1-CDEb7). This might lead to the application of CDEb in the nutritional, pharmaceutical and cosmetic products directly. Two active compounds, 7-*O*-R-apigenin and hydroquinone, were firstly reported from Thai bee pollen. Further investigations should be performed to obtain more active compounds. The relative composition of individual active compounds affecting the free radical scavenging activity should be studied.

## Competing interests

The authors declare that they have no competing interests.

## Authors’ contributions

AC collected the sample, prepared the extracts and carried out the experiments. PP contributed to the study design, analyzed and interpreted the data. KK, MO, HM and AK contributed to invaluable suggestions and comments on research. CC did the design and supervision of the experiments and contributed to drafting the manuscript. All authors read the manuscript, contributed in correcting it and approved its final version.

## Pre-publication history

The pre-publication history for this paper can be accessed here:

http://www.biomedcentral.com/1472-6882/12/45/prepub
